# Community perspectives on food insecurity and obesity: Focus groups with caregivers of metis and Off-reserve first nations children

**DOI:** 10.1186/s12939-015-0232-5

**Published:** 2015-10-16

**Authors:** Jasmin Bhawra, Martin J. Cooke, Rhona Hanning, Piotr Wilk, Shelley L. H. Gonneville

**Affiliations:** School of Public Health and Health Systems, University of Waterloo, Waterloo, ON Canada; Department of Sociology and Legal Studies, University of Waterloo, Waterloo, ON Canada; Schulich School of Medicine and Dentistry, Western University, London, ON Canada; Métis Nation of Ontario, Ottawa, ON Canada

**Keywords:** Food insecurity, Aboriginal peoples, First nations, Métis, Child obesity, Canada, Income

## Abstract

**Introduction:**

Aboriginal children in Canada are at a higher risk for overweight and obesity than other Canadian children. In Northern and remote areas, this has been linked to a lack of affordable nutritious food. However, the majority of Aboriginal children live in urban areas where food choices are more plentiful. This study aimed to explore the experiences of food insecurity among Métis and First Nations parents living in urban areas, including the predictors and perceived connections between food insecurity and obesity among Aboriginal children.

**Methods:**

Factors influencing children’s diets, families’ experiences with food insecurity, and coping strategies were explored using focus group discussions with 32 parents and caregivers of Métis and off-reserve First Nations children from Midland-Penetanguishene and London, Ontario. Four focus groups were conducted and transcribed verbatim between July 2011 and March 2013. A thematic analysis was conducted using NVivo software, and second coders ensured reliability of the results.

**Results:**

Caregivers identified low income as an underlying cause of food insecurity within their communities and as contributing to poor nutrition among their children. Families reported a reliance on energy-dense, nutrient-poor foods, as these tended to be more affordable and lasted longer than more nutritious, fresh food options. A lack of transportation also compromised families’ ability to purchase healthful food. Aboriginal caregivers also mentioned a lack of access to traditional foods. Coping strategies such as food banks and community programming were not always seen as effective. In fact, some were reported as potentially exacerbating the problem of overweight and obesity among First Nations and Métis children.

**Conclusion:**

Food insecurity manifested itself in different ways, and coping strategies were often insufficient for addressing the lack of fruit and vegetable consumption in Aboriginal children’s diets. Results suggest that obesity prevention strategies should take a family-targeted approach that considers the unique barriers facing urban Aboriginal populations. This study also reinforces the importance of low income as an important risk factor for obesity among Aboriginal peoples.

## Introduction

Child obesity is an urgent public health issue in Canada and other wealthy countries. The World Health Organization defines obesity as the accumulation of excess fat to the point where it has adverse impacts on health [1]. Approximately one third of Canadian children between the ages of 5 to 17 years could be considered overweight or obese, with some populations at an even higher risk [2]. Aboriginal children are disproportionately affected by obesity, as they are twice as likely to be classified as obese compared to their non-Aboriginal Canadian counterparts [3, 4]. This is true for children of each of the three Aboriginal groups identified in the 1982 *Constitution Act* – First Nations, Métis and Inuit – which together make up about four per cent of the Canadian population or 1.5 million people [5], and for those living in urban areas as well as in rural or remote First Nations or Inuit communities. Although obtaining a comparable measurement of child obesity among Aboriginal children can be problematic, an estimated 20 % of First Nations children living off reserve^1^ (aged 5 to 17), and 16.9 % of Métis children could be classified as obese [4], compared with 11.7 % of Canadian children aged 5 to 17 [2].

The poorer average health of Aboriginal peoples, relative to other citizens in wealthy former colonies including Canada, the United States, Australia and New Zealand [6], as well as poorer ones in South and Central America and elsewhere in the “new world” has been well documented [7]. Although colonization progressed differently in these countries and the colonists differed somewhat in their orientations to Aboriginal peoples, including in the degree of violence employed in subjugating them, the overarching logic of colonialism was the same. This included dispossession from traditional lands and waters, devaluation of indigenous languages and cultures, and attempts either to assimilate dominated people to settler economic systems or to relegate them to marginal “reserve” lands [8].

In Canada, the mechanisms of colonialism have included the *Indian Act*, which continues to legally define and fragment Aboriginal peoples, and residential schooling, which led to generations of Aboriginal children being raised in institutions and away from their families [8]. Colonial institutions and practices have had different implications for each of the three recognized Aboriginal groups in Canada. “First Nations,” an umbrella term referring to about a dozen major linguistic and cultural groups living across the continent below the Arctic, have been the subjects of the *Indian Act* and reserve system, resulting in a situation in which about half of those identifying as First Nations are legally Registered Indians, with roughly 60 % of those living in First Nations reserves. Somewhat paradoxically, Métis have suffered from *not* being recognized as an Aboriginal people. The result of a historical blending of European and First Nations cultures, mainly around the fur trade in Western Canada, Métis ethnogenesis and the development of a distinct culture and language have been identified as occurring before Canadian confederation in 1867 [9]. For most of their history, Métis were recognized neither as an Aboriginal people nor as fully European/Canadian, resulting in exclusion from treaties and land settlements and, until recently, from Aboriginal hunting and gathering rights. Inuit, the people of the North, were the last to be fully colonized. After the Second World War, the federal government pursued an active program of assimilation of Inuit [10], and in recent decades economic activity around resource development in the North have further contributed to many Inuit finding themselves culturally and economically dislocated, neither able to practice a traditional way of life nor integrated into a modern wage economy.

The results of historical and contemporary colonial practices can be seen today in ongoing disparities in income, educational attainment and labour force participation, each of which are considered important “upstream” determinants of population health [11]. Although the income gaps narrowed somewhat in the 1990s, First Nations Inuit and Métis continue to have significantly lower average incomes than non-Aboriginal Canadians, and this is true for the more than half of the population that currently lives in cities^2^, as well as those who live in First Nations reserves or Inuit communities [12].

The relationships between income inequalities, other social determinants, and particular health outcomes are complex, and may involve material standards of living, psychosocial effects, social capital, and other pathways [13]. In the particular case of obesity among Aboriginal children, Willows Hanley and Delormier (2012) have proposed a socioecological framework to understanding these effects [14]. At the most proximate level, children’s weights are affected by their energy intake and expenditure, mediated by genetic factors. However, children’s diets and physical activities are themselves shaped by family characteristics, including incomes, knowledge about healthy eating and exercise, and parents’ behaviours. These are further affected by factors associated with neighbourhood and communities, such as access to safe play spaces and the local cost of food, or the availability of recreation programs. All of these can be placed within the context of macro-level social and economic structures, including the industrial production of food and the wage economy. Critically, in the case of Aboriginal peoples, this macro context includes colonialism and continued social and economic marginalization [14].

The research reported in this paper is part of a broader project to understand some of the specific mechanisms that produce the excess risk of obesity among Aboriginal children to inform the design of public health interventions to reduce this risk. In this paper we focus on the role of “food security” in this respect. Aboriginal peoples in Canada are at a higher risk for food insecurity, generally defined as the limited or uncertain availability of nutritionally adequate and safe foods [15, 16, 17]. This is most obviously the case for rural and remote First Nations reserves and Inuit communities, in which the high cost of fresh foods transported from the south has combined with decreasing use of traditional food sources to result in diets characterised by consumption of inexpensive, but nutritionally poor, packaged foods [18, 19]. However, urban First Nations and Métis, who have not been part of the reserve system, have higher risk for food insecurity as well as for obesity. Aboriginal households in urban areas, and also in non-remote rural areas, have a risk of food insecurity that is up to three times that of non-Aboriginal households [8, 17].

Much of this higher prevalence of both food insecurity and obesity is certainly due to higher rates of poverty among Aboriginal peoples [20]. Lower household income has been found to be related to a higher risk of obesity among Métis children [21], and neighbourhood income has been related to child obesity among Canadian children in general [22]. The specific mechanisms that connect food insecurity and child obesity are not necessarily obvious, though, nor are the options for addressing these issues. Among low-income Canadians in general, nutritional knowledge and food skills have been suggested as possible factors connecting income to nutrition, although the evidence indicates that these effects might not be significant [23], and that it is income adequacy that is the critical factor [24]. When considering the nutrition of children, mothers’ strategies for mitigating the effects of food insecurity may include reducing their own consumption, in addition to the use of food banks, community gardens, and community kitchens [25]. There is evidence, however, that those community-based services are under-utilized by the food insecure and are therefore not reaching the intended populations [26].

In the case of Aboriginal populations, the connections are even less clear. A central consideration in the definition of food insecurity should be the cultural appropriateness and acceptability of available food, not just its affordability [27]. For Métis and First Nations people in urban areas, the experience of food insecurity, food preferences, coping strategies, and the perceived connections between the food environment and children’s health may be different from those of non-Aboriginal Canadians or from those living in discrete Aboriginal communities. Appropriate strategies for addressing these issues may also be different, and should not simply be extrapolated from other contexts.

This study helps to address the lack of previous qualitative research on food insecurity and obesity among Métis and urban First Nations children. It was conducted in the context of a broader mixed-methods study of the determinants of child obesity among Aboriginal children, with the intention of identifying promising directions for community-based programming to reduce obesity risk^3^. Using semi-structured focus groups with parents and caregivers of Métis and First Nations children, we explored parents’ perceptions of the factors that affected the ability of families to provide culturally appropriate and healthful food, and the strategies that community members used to address food insecurity. We draw conclusions regarding the connections between food insecurity and child obesity in this population, and propose some avenues for further research.

## Methods

### Study design

A total of four focus groups were conducted with First Nations and Métis caregivers in London and Midland-Penetanguishene, respectively. Two focus groups were held in Midland-Penetanguishene in partnership with the Métis Nation of Ontario, and the latter two focus groups took place in London in collaboration with a local Aboriginal health centre. These locations were chosen because they are urban settings with relatively large First Nations and Métis populations, but represent different geographic contexts.

**Fig. 1:**
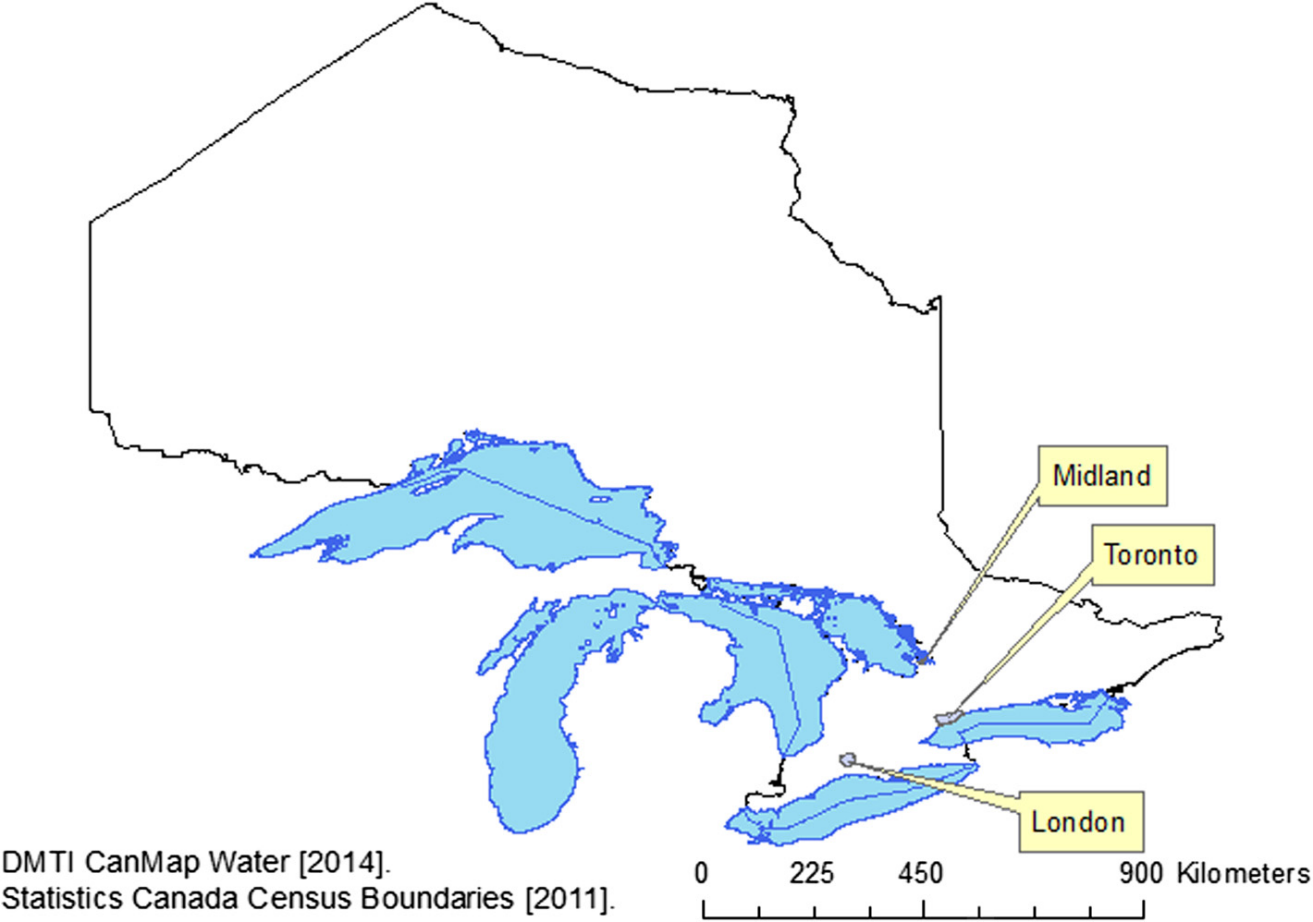
Location of London and Midland, Ontario

London is a medium-sized Canadian city located in southwestern Ontario with a population of 467,225 people in 2011 [28]. It is located close to several First Nations reserves and is approximately halfway between Toronto, Ontario and Detroit, Michigan. According to the 2011 National Household Survey, 6200 people living in the city identified themselves as First Nations, and approximately 2000 as Métis [28] (Fig. 1).

Midland-Penetanguishene, which includes the Town of Midland and several nearby townships, is a more rural community located in Northern Ontario with a total population of approximately 43,000 people in 2011 [29, 30]. It experiences seasonal tourism, which brings upwards of 100,000 visitors in the summer months. Métis people are 11 % of the population (4800 people) in Midland-Penetanguishene making it a major concentration of Métis in the province. Approximately 1500 residents identified themselves as First Nations in the 2011 National Household Survey [29, 30].

Purposive, convenience sampling was used in collaboration with local Aboriginal partner organizations. Recruitment was conducted using advertisements in local newspapers as well as direct referrals by collaborating organizations. The convenience sampling method was appropriate for this group, as existing rapport and trust were important to ensuring that potential respondents felt comfortable sharing their insights with our research group. Parents or caregivers of Métis or First Nations children under 18 were invited to participate in the focus group sessions. The recruitment referred to “parents and caregivers”, as opposed to only parents, because Aboriginal children are more likely to live in intergenerational households and parents may not be the primary guardians [31, 32], and we therefore use “caregivers” in the remainder of the paper. Income level, food security, and child weight status were not used as inclusion criteria in order to capture the broad range of respondents’ experiences, promote inclusivity, and because of the sensitive nature of the topics discussed.

### Participants and procedure

A total of 32 caregivers were interviewed during the 90-minute sessions, with each focus group ranging from 5 to 11 participants. As suggested by collaborating organizations, the discussions were opened by an Elder or spiritual leader, who remained present for the session to offer support should any difficult or upsetting issues be brought up. All sessions were led by the same experienced Indigenous facilitator. The interview guide and focus group procedure were reviewed and approved by the research ethics boards at the University of Waterloo and Western University.

The focus groups were held in July 2011 and December 2011 in Midland-Penetanguishene, and December 2012 and March 2013 in London, Ontario. Information and consent forms were administered prior to the focus groups, and background questionnaires were completed following the focus groups to obtain basic demographic information and provide an opportunity for additional feedback.

The interview guide included questions about the health of children in the community, as well as barriers and facilitators to healthy eating and physical activity. Questions were kept open ended and phrased to capture community-level issues and examples, however the majority of participants drew from personal experiences during the discussions.

### Data analysis and qualitative rigor

All focus groups were audio recorded and professionally transcribed. Participants’ identities were concealed throughout the data analysis and care was taken to ensure that identities could not be revealed in the quotations selected for reporting results. Participants were given the option to withdraw from the focus groups at any time. A thematic analysis was conducted involving coding individual transcripts into major themes using NVivo qualitative data analysis software Version 10 (2013) [33]. Codes were created for distinct ideas or concepts and organized into a coding manual. Transcripts were then uploaded into NVivo, and codes from the manual were entered as nodes into the program. This process allowed for reoccurring, prominent themes to be identified and examined within and across focus groups.

Experiences with food insecurity, coping strategies, as well as barriers and facilitators to healthy eating were themes of particular interest. While the coding manual was initially organized based on *a priori* research questions and concepts, including the barriers and facilitators to healthy eating, the content of the coding manual was modified as the analyses proceeded, and the research questions provided a general focus for conducting the analysis instead of a specific set of expectations or findings.

The next step involved testing the reliability and dependability of these codes to ensure rigor in this qualitative study. A reliability check was conducted using second coders. After the initial coding and coding manual development, two additional coders were provided with a copy of the coding manual and asked to re-code all transcripts. If a code was missing or inappropriately assigned, the second coders modified the manual accordingly. After debriefing with the second coders, a consensus was reached on all codes, which were then entered into NVivo. Dependability of the results was calculated using inter-rater reliability. The inter-rater reliability between the first and second coders was 88 % for the focus groups in Midland-Penetanguishene and 85 % in London, which surpasses the minimum requirement of 70 % suggested by Miles and Huberman (1994) [34]. Following the data analysis, reports were produced for the collaborating Métis and First Nations organizations to ensure the applicability of the findings and appropriateness of interpretation.

## Results

Twenty-three caregivers of Métis children and nine caregivers of First Nations children participated in the focus group discussions. The majority of respondents were women (81 %). Demographic characteristics from the two focus groups can be found in Tables 1 and 2.

**Table 1:** Demographic Characteristics of First Nations Caregivers from Focus Groups in London, Ontario

	Number	Percent
Gender		
Male	1	11 %
Female	8	89 %
Total participants	9	100 %
Years lived in the community		
Less than 1	0	0 %
1 to 5	2	22 %
6 to 10	1	11 %
11 to 19	1	11 %
20 to 25	1	11 %
30 to 34	2	22 %
35 to 39	0	0 %
40 +	1	11 %
Total Participants	8^a^	100 %^a^

The background questionnaire in London was slightly revised by a partnering Aboriginal organization, hence does not include questions about number of children living in the household. Only a few participants answered the question about children’s ages, hence this question was omitted in the table as well. The 25 to 30 year category for total number of years in the community is missing.

^a^One participant was living on reserve just outside of London at the time of the interview

**Table 2:** Demographic Characteristics of Métis Caregivers from Focus Groups in Midland-Penetanguishine, Ontario

	Number	Percent		Number	Percent
Gender			Number of children in the household		
Male	5	22 %	One	6	30 %
Female	18	78 %	Two	5	25 %
Total	23	100 %	Three	3	15 %
Years lived in the community			None/no response	6	30 %
Less than 1	2	9 %	Total Households	20	100 %
1 to 5	2	9 %			
6 to 10	2	9 %	Ages of children in the household		
11 to 20	2	9 %	1 to 4	5	19 %
20 to 25	2	9 %	5 to 9	3	12 %
30 to 34	6	26 %	10 to 13	5	19 %
35 to 39	2	9 %	14 to 16	7	27 %
40 +	5	22 %	17 to 23	6	23 %
Total Participants	23	100 %	Total children	26	100 %

The thematic analysis of respondents’ statements provided detailed insight into community members’ challenges with healthy eating and coping strategies. Two key barriers to children’s healthy diets that were raised by respondents were low income and difficulty in physically accessing fresh foods. It is important to note that the facilitators did not present income inadequacy and food insecurity as topics, rather they were brought up by participants as key factors which affected Aboriginal parents’ ability to provide nutritious food for their families. The availability of traditional foods was also raised as a concern. Participants also discussed various coping strategies for dealing with food insecurity, and identified ways that these strategies might be leading to the risk of obesity among Aboriginal children. These main themes are described briefly below.

### Low income as a main determinant

A majority of participants felt that the unaffordability of healthy food was an overarching barrier to improving children’s diets. Several caregivers regarded healthy food options to be too costly, especially as compared to processed or convenience foods that were at a lower cost. A caregiver from Midland-Penetanguishene said:

So, you know, the fattier foods are the lowest price and they go a lot further. So, you’re going to see obesity in that stereotypical low-income/one income family. And if you take a two-income family, yes, you know what, there’s more money coming in. So, yes, they can get the fresh fruits, they can get the fresh vegetables, they can buy the milk, they can, you know, they don’t have to live on Kraft Dinner and soup.

For some families, the issue was not necessarily the cost of healthy food in particular, but rather that food in general was unaffordable. However, some respondents indicated that families who could afford more healthy food might still consume “junk” food. A First Nations caregiver stated:

So yeah, it depends on the parents, if they’re working they can afford more food for their kids and then they buy both food, like the health food and the junk food at the same time.

### Accessibility and transportation

Although neither Midland-Penetanguishene or London are remote communities and both are well-served by grocery and other food outlets, parents from both communities described difficulty with physically accessing healthy food for their households and how this negatively affected their food choices and children’s diets. Problems with accessibility were often related to income. Several participants described their reliance on public transportation for grocery shopping, and noted that some grocery stores were located in areas that were difficult to access via public transit. Some Midland Métis parents mentioned that accessibility was a particular problem for families living in some parts of the area that were poorly served by public transport in general. One First Nations caregiver mentioned that inconvenient access to grocery stores impacted the frequency of her shopping trips:

Yeah, that’s what we do because we don’t have a car so we go, like my mom will drive me to the grocery store once a month and then if it [food] goes bad, it goes bad and we just have to wait until next month.

Less frequent grocery shopping also affected food-purchasing behaviour. Oftentimes non-perishable food items were a more economical choice since these foods lasted longer than fresher options. Caregivers acknowledged that non-perishable items tended to be the least nutritious. A First Nations caregiver stated:

I think it’s because sometimes when you buy fruits and vegetables, they tend to, like the shelf-life is not as long as the other foods. We just recently moved to the reserve [adjacent to the city] and you need a vehicle to get to town and buy those foods like every so often […], and a lot of people that live on the reserve, they go grocery shopping maybe once or twice a month, so they’re not able to continuously get fruits and vegetables.

### Access to traditional foods

In general, caregivers felt that a shift away from more traditional First Nations or Métis diets to a “Western” diet was an important contributing factor to overweight and obesity among their children. Traditional Aboriginal diets were considered nutrient rich compared to children’s current diets, which were described using various terms (i.e., “packaged foods”, “high carbohydrate”, “junk food”), as energy-dense and nutrient-poor.

There were several aspects to the lack of traditional foods in contemporary diets. Several mentioned children’s preferences for Western food over wild game, for example. Some caregivers believed that this was because children were not given enough opportunity to develop a preference for traditional foods due to the challenges with accessing and affording these options.

When asked about the availability of traditional food items in their communities, caregivers said that these foods were too expensive or simply unavailable, hence introducing Aboriginal foods at home was a challenge. Although Midland-Penetanguishine is located near what would have been traditional harvesting regions, one Métis caregiver said:

You can get it, but it’s expensive. And not all of the traditional foods are easy to get, like wild game, is not easy to get.

Caregivers felt that one reason for the lack of traditional food in contemporary diets was because of the decline in hunting, trapping, fishing, or gathering activities among the community members themselves. Another reason was the change in communal food practices. Some First Nations caregivers mentioned that the traditional practices regarding sharing food among community members had declined and were not practiced in the city. Others mentioned that knowledge of traditional foods and preparation was endangered, and that young parents, in particular, might not be able to prepare traditional meals for their children, even if the foods were available.

Although they were not common, there were some concerns expressed about the quality of some wild game that could be obtained. In London, one parent indicated that she would not consume the fish caught in the local river because of the fears that it had been contaminated by pollution.

Some community members also pointed out that foods thought of as “traditional” were problematic. “Fry bread” (a deep-fried dough) and “Indian Tacos” (tacos made with fry bread) were mentioned by some First Nations participants as foods that had become associated with First Nations community gatherings, but were neither “traditional” in the sense of being part of First Nations diets before European contact, nor healthy.

### Coping strategies for food insecurity

In order to deal with food insecurity and not having enough money for healthy food, caregivers spoke about several different coping strategies. Some caregivers mentioned borrowing money or sharing food as options, however the more commonly identified strategies involved relying on family and community programming.

Parents and caregivers mentioned many different food programs offered in London and Midland-Penetanguishene, including food box programs, food banks, soup kitchens, school breakfast or lunch programs, church meal programs, community kitchens, community gardens, prenatal nutrition programs, as well as programs on reserve that were used by some off-reserve First Nations residents of London. Caregivers reported resorting to these programs if there was not enough money for food or food at home, and sometimes for obtaining healthy food options. One participant described a program at his community church:

I used to call my church in my old neighbourhood, because I just moved recently, and I would go there and they would help me out with a grocery card. You’re allowed to go there every 3 months, but I would go there about maybe once or twice a year when I needed to.

However, participants identified numerous barriers to programming that either hindered their participation or the programs’ effectiveness. Caregivers specifically spoke about the food box programs and food banks within their communities. They felt that these programs often had food of poor quality that was either near to or past its expiration date. In addition to subpar food options, many caregivers from Midland-Penetanguishene recalled feelings of shame associated with using food banks:

It takes a lot to swallow your pride to access these resources, and if you’re gonna go there and be judged by the person that’s there that’s supposed to be helping you, you know, it’s gonna be harder to swallow your pride next time. And we are a small community we know people, and you walk through a door and your neighbours are sitting there at the table volunteering.

The feelings of judgment exacerbated the stigma caregivers already felt were associated with receiving food charity. A caregiver from London reflected that stigma in her comments:

I think a lot of people take advantage of that, too, the free stuff, the free food and everything. The way I see it is a lot of the drug users and alcoholics, they can just use their cheque and blow it because they’re going to get free stuff. That’s the way I see it because it seems like there’s like, I don’t know a lot of people think that, or talk about it in a way that it’s just the people that use drugs or alcoholics that go to these free things all the time, but it’s not. But then when people talk about it, that’s the way they talk about things.

Some parents also noted that the types of fresh foods sometimes provided by food banks or “good food boxes” might require cooking or preparation skills that community members might not have. Describing a woman given a bag of potatoes, a First Nations woman commented:

She didn’t know what to do with it. And some people wouldn’t know how to cook [them] so it’s that sort of mindset as well that you’re not used to having these sorts of foods that you don’t know what to do with them.

When asked how community programming could be made more effective, caregivers described several strategies including shifting the focus from the child to a more family-oriented approach:

I think a lot of focus is put into child health here and there whatever, but if more focus was put on […] the family, promoting more family unit type activities where all took part, whatever their abilities are. I think that would make good, positive change.

Additionally, caregivers felt that programs with an Aboriginal cultural component would encourage overall health for children and their communities:

I think we need more, um, […] Aboriginal days for example, that when you go there everybody in the community is welcome, not only Métis people. We might not feel comfortable going to something that is mainstream, but mainstream people may not necessarily […] it’s that education part again. If we have more community/family things that we have multiple opportunities to try different foods that are healthy and engage with different people.

Overall, caregivers identified numerous barriers faced by families to provide healthy foods for their children. Some of these barriers were exacerbated by underlying problems such as low income within the household, which strongly impacted accessibility and food purchasing options.

## Discussion

This qualitative study helps us to understand the connections between food insecurity and children’s diets, and uncovers the importance of income, food security, and coping strategies for the weight status of Aboriginal children. Community members’ perspectives elucidate the lived experience of Aboriginal families in urban settings, and how the issue of food insecurity continues to be prevalent even among children living alongside sizable non-Aboriginal populations.

The focus group discussions describe a clear relationship between low income and food insecurity, which participants believed had an adverse impact on their children’s diets. Current diet practices were perceived to be an important contributor to high obesity rates among First Nations and Métis children. While caregivers did not use the language, “food insecurity,” conversations about not having enough food or money for food, and strategies for coping with these conditions, suggest that food insecurity was present and manifests itself in different ways.

Caregivers discussed poor variety of foods, compromised fruit and vegetable intake, as well as the shift away from traditional foods as examples of how food consumption and purchasing patterns changed with food security status. Food insecurity had a negative impact on children’s diets, and many caregivers attributed the rise in overweight and obesity to poor diet quality. The wide range of barriers and facilitators to healthy eating and community programming illustrate some potential opportunities for intervention.

It was somewhat unexpected that accessibility of grocery stores would be a major barrier to healthy eating in the communities studied. Accessibility is often discussed in the literature as a barrier for families living in geographically remote settings or on reserve [35, 36]. In both Midland-Penetanguishene and London, caregivers spoke about difficulties accessing public transit, as well as grocery stores being inconveniently located. However it is important to note that convenience and location were not the main hindrances to healthy food access, rather it was low income that made accessing grocery stores so inconvenient. Many caregivers relied on public transit because they could not afford a car, hence the length or distance of grocery stores trips were affected as a result. Despite living in urban settings where the risk of food insecurity should be relatively lower than geographically remote areas, caregivers’ experiences clearly demonstrated that food insecurity persists in these households.

The focus groups confirmed that, at least in the views of the participants, low income and resulting food insecurity are likely to be implicated in the higher risk of obesity among Aboriginal children. The community programs that were mentioned as coping strategies by participants tended to address immediate individual needs, rather than the systematic or structural factors leading to low income, and did not appear to be effective in meeting those needs. While community nutrition programs played an important role in facilitating the accessibility and affordability of healthy foods, caregivers described numerous barriers that affected the effectiveness and outreach of those programs. Although food banks helped ensure an adequate quantity of food within households, the quality was seen as often poor and not culturally appropriate; hence they did not seem to contribute to the healthfulness of families’ diets. It was also important to learn that caregivers were not comfortable visiting food banks because of the stigma associated with food charity. This deterred caregivers even in times of need because they felt that they were being discriminated by the volunteers and also ashamed for needing to use the food bank. Although many people report shame associated with using food banks, this stigma is perhaps worse for Aboriginal peoples [37].

Successfully and permanently reducing the health inequities experienced by Aboriginal peoples likely requires a change in the macro-level structural factors that are the fundamental causes of those inequities. In Canada, as elsewhere, these are most likely to be addressed by Aboriginal peoples’ political action. However, we also think that local public health programming may play a role in reducing insecurity and therefore the risk of obesity among children. Despite the demonstrated higher risk, there have been relatively few programs or interventions aimed at reducing obesity specifically among Aboriginal children, and most have been conducted in discrete Aboriginal communities rather than in urban areas [38].

Many of these existing programs have targeted changing children’s physical activity and eating behaviours. However, the group interviews suggested that effective programming might focus more on addressing the applied skills that are necessary for eating healthy, such as meal preparation and preservation, harvesting, food storage and cooking. Many of these skills may have been lost through residential schooling or lack of access to traditional activities. Programming with a strong cultural component might have more meaningful impact, and caregivers suggested taking a more family-targeted approach rather than placing sole emphasis on children. Importantly, caregivers discussed the importance of healthy living overall because they believed it had the potential to address a wide range of health problems for children in the community. Successful community interventions therefore might take a more holistic approach to health, rather than focusing on a single problem such as obesity.

### Study limitations

Child obesity and food insecurity are both sensitive topics that had to be carefully approached during the focus group discussions. The discussions did not probe too deeply into families’ coping strategies and personal experiences of food insecurity to avoid making participants feel uncomfortable. However the facilitator and interview guide questions were still able to obtain important and relevant information on the topic. While one-on-one interviews could have been better for accessing more personal information, focus groups were still a better fit given that the objective was not to gain an understanding of the individual experiences, instead the point was to get a broader understanding of what families and children experienced within those communities. Focus groups allowed caregivers to comment beyond their personal experience and share what they had observed in the community. Additionally, the use of an Indigenous facilitator increased participants’ comfort with sharing their views, and many drew from personal experience.

Since the focus groups took place in only two Aboriginal communities in Ontario, results are not generalizable. Although it is important to note that the results are not intended to be generalizable to all Métis and First Nations, or to Aboriginal peoples in general. Instead, they provide some insight into the experience of food insecurity and implications of low-income in urban settings for child health.

## Conclusion

Few studies have explored the effects of food insecurity on obesity, and none have focused on Aboriginal children living in urban settings despite the fact that they are among the most severely impacted by these two health concerns. There have also been limited studies exploring this topic using qualitative research methods. The focus group discussions around barriers and facilitators to healthy eating, as well as how these barriers relate to obesity, allowed for the identification of some of the challenges that face Aboriginal families living in urban areas.

The results indicate that these challenges may include issues such as a lack of transportation and access to healthy food, which are related to the underlying problem of low income, but are also exacerbated by local conditions such as a lack of public transportation. A lack of access to traditional foods may be a particular problem for people in urban communities. The results support the idea that interventions to reduce the risk of obesity among urban Aboriginal children may benefit from a focus on improving families’ food security, and that those efforts should also consider the unique contexts in which those families live.

Future research should focus on exploring food insecurity and diet quality to better understand the relationship with obesity rates among Aboriginal children. Additional focus group discussions are important to identifying strategies to improve program design and delivery. Larger focus groups would also enable a comparison between Aboriginal communities living in different geographic settings. Overall, caregivers and community members provide valuable insights and expertise regarding barriers and facilitators to their children’s health and should be included in conversations regarding Aboriginal-focused program planning and policy.

## Footnotes

1 “Reserves” are Crown lands set aside for the use of First Nations. Métis and Inuit peoples have not historically been part of the reserve system.

2 In 2011, 75 % of Status First Nations, 42 % of non-Status First Nations, 71 % of Métis, and 43 % of Inuit lived in urban areas [5]

3 This research was funded by the Canadian Institutes for Health Research, Institute for Aboriginal Peoples’ Health Operating Grant RN125827–251526

## References

[1] World Health Organization (WHO). Obesity. *WHO* 2013, [http://www.who.int/topics/obesity/en/]

[2] Roberts KC, Shields M, de Groh M, Aziz A, & Gilbert J. Overweight and obesity in children and adolescents: Results from the 2009 to 2011 Canadian Health Measures Survey (Catalogue no. 82-003-XPE). *Statistics Canada.* 2012. [http://www.statcan.gc.ca/pub/82-003-x/2012003/article/11706-eng.pdf] 23061263

[3] Shields, M. Measured obesity: Overweight Canadian children and adolescents. (Catalogue no. 82-620-MWE2005001). *Statistics Canada.* 2005, [http://www5.statcan.gc.ca/access_acces/archive.action?loc=/pub/82-620-m/2005001/pdf/4193660-eng.pdf]

[4] Public Health Agency of Canada (PHAC): Obesity in Canada: A joint report from the Public Health Agency of Canada and the Canadian Institute for Health Information. (Catalogue no. HP5-107/2011E-PDF). *PHAC.* 2011, [http://www.phac-aspc.gc.ca/hp-ps/hl-mvs/oic-oac/index-eng.php]

[5] Aboriginal Affairs and Northern Development Canada (AANDC). Aboriginal Demographics from the 2011 National Household Survey. *AANDC.* 2013, [https://www.aadnc-aandc.gc.ca/eng/1370438978311/1370439050610]

[6] Cooke M, Mitrou F, Lawrence D, Guimond E, Beavon D (2007). Indigenous well-being in four countries: an application of the UNDP’s Human Development Index to Indigenous Peoples in Australia, Canada, New Zealand and the United States. BMC Health Hum Rights.

[7] Gracey M, King M (2009). Indigenous health part 1: determinants and disease patterns. Lancet.

[8] King M, Smith A, Gracey M (2009). Indigenous health part 2: the underlying causes of health. Lancet.

[9] Adams C, Dahl G, Peach I (2013). Métis in Canada: History, identity, law and politics.

[10] Bonesteel, J. Canada’s Relationship with Inuit: a history of policy and program development. *Statistics Canada.* 2006, [http://www.aadnc-aandc.gc.ca/eng/1100100016900/1100100016908]

[11] Mitrou F, Cooke M, Lawrence D, Povah D, Mobilia E, Guimond E (2014). Gaps in indigenous disadvantage not closing: a census cohort study of social determinants of health in Australia, Canada, and New Zealand from 1981–2006. BMC Public Health.

[12] Pendakur K, Pendakur R (2011). Aboriginal income disparity in Canada.

[13] Evans W, Wolfe B, Adler N (2012). The SES and health gradient: a brief review of the literature.

[14] Willows ND, Hanley AJ, Delformier T (2012). A socioecological framework to understand weight-related issues in Aboriginal children in Canada. Appl Physiol Nutr Metab.

[15] Anderson SA (1990). Core Indicators of Nutritional State for Difficult-to-Sample Populations. J Nutr.

[16] Willows ND, Veugelers P, Raine K, Kuhle S (2009). Prevalence and sociodemographic riskfactors related to household food security in Aboriginal peoples in Canada. Public Health Nutr.

[17] Health Canada. Household Food Insecurity In Canada in 2007–2008: Key Statistics and Graphics*. Health Canada.* 2012, [http://hc-sc.gc.ca/fn-an/surveill/nutrition/commun/insecurit/key-stats-cles-2007-2008-eng.php#fnb1]

[18] Haman F, Fontaine-Bisson B, Batal M, Imbeault P, Blais JM, Robidoux MA (2010). Obesity and Type 2 Diabetes in Northern Canada’s Remote First Nations Communities: The Dietary Dilemma. Int J Obes.

[19] Chan HM, Fediuk K, Hamilton S, Rostas L, Caughey A, Kuhnlein H, Egeland G, Loring E (2006). Food security in Nunavut, Canada: barriers and recommendations. Int J Circumpol Health.

[20] Health Canada. Canadian Community Health Survey, Cycle 2.2, Nutrition (2004): Income-related household food security in Canada. *Health Canada.* 2007, [http://www.hc-sc.gc.ca/fn-an/surveill/nutrition/commun/income_food_sec-sec_alim-eng.php#fg33]

[21] Cooke MJ, Wilk P, Paul KW, & Gonneville SLH. Predictors of obesity among Métis children: Socio-economic, behavioural, and cultural factors. *CJPH.* 2013, 104. [http://journal.cpha.ca/index.php/cjph/article/view/3765/2833] 10.17269/cjph.104.3765 PMC6973954 24044469

[22] Oliver LN, & Hayes MV. Effects of neighbourhood income on reported body mass index: an eight year longitudinal study of Canadian children. *BMC Public Health.* 2008, [http://www.biomedcentral.com/1471-2458/8/16] 10.1186/1471-2458-8-16 PMC2291462 18194577

[23] Power, EM. Determinants of healthy eating among low-income Canadians. *CJPH.* 2005, 96. [http://journal.cpha.ca/index.php/cjph/article/view/1504/1693] 10.1007/BF03405200 PMC6975906 16042163

[24] Kirkpatrick S, Tarasuk V (2003). The relationship between low income and household food expenditure patterns in Canada. Public Health Nutr.

[25] McIntyre L, Glanville T, Raine KD, Dayle JB, Anderson B, Battaglia N (2003). Do low-income lone mothers compromise their nutrition to feed their children?. CMAJ.

[26] Kirkpatrick SI, & Tarasuk V. Food insecurity and participation in community food programs among low-income Toronto families. *CJPH.* 2009, 100. [http://journal.cpha.ca/index.php/cjph/article/view/1771/1955] 10.1007/BF03405523 PMC6973985 19839291

[27] Power EM (2008). Conceptualizing food security for Aboriginal people in Canada. CJPH.

[28] Statistics Canada. NHS Focus on Geography Series–London. *Statistics Canada.* 2014, [http://www12.statcan.gc.ca/nhs-enm/2011/as-sa/fogs-spg/Pages/FOG.cfm?lang=E&level=3&GeoCode=555]

[29] Statistics Canada. NHS Focus on Geography Series– Midland. http://www12.statcan.gc.ca/nhs-enm/2011/as-sa/fogs-spg/Pages/FOG.cfm?lang=E&level=3&GeoCode=571.

[30] Statistics Canada. NHS Focus on Geography Series– Penetanguishene. http://www12.statcan.gc.ca/nhs-enm/2011/as-sa/fogs-spg/Pages/FOG.cfm?lang=E&level=4&GeoCode=3543072.

[31] O’Donnell V, & Wallace S. First Nations, Métis and Inuit Women. *Statistics Canada.* 2011, [http://www.statcan.gc.ca/pub/89-503-x/2010001/article/11442-eng.htm#a15]

[32] Statistics Canada. 2011 National Household Survey: Aboriginal Peoples in Canada. *Statistics Canada.* 2011, [http://www.statcan.gc.ca/daily-quotidien/130508/dq130508a-eng.pdf]

[33] NVivo qualitative data analysis software. QSR International Pty Ltd. Version 10. 2013.

[34] Miles MB, Huberman AM (1994). *Qualitative data analysis: An expanded sourcebook*.

[35] Elliot B, Jayatilaka D, Brown C, Varley L, & Corbett KK. “We are not being heard”: Aboriginal perspectives on traditional foods access and food security. *J Environ Public Health.* 2012. [http://www.hindawi.com/journals/jeph/2012/130945/] 10.1155/2012/130945 PMC3549364 23346118

[36] Willows ND (2005). Determinants of healthy eating in Aboriginal peoples in Canada: the current state of knowledge and research gaps. Can J Public Health.

[37] Hamelin A, Beaudry M, Habicht J (2002). Characterization of household food insecurity in Quebec: food and feelings. Soc Sci Med.

[38] Towns C, Cooke M, Rysdale L, Wilk P (2014). Healthy weights interventions in Aboriginal children and youth: a review of the literature. Can J Diet Pract Res.

